# Novel mutations in a second primary gastric cancer in a patient treated for primary colon cancer

**DOI:** 10.1186/s12957-023-03057-y

**Published:** 2023-06-07

**Authors:** Roli Purwar, Madhumita Tripathi, Monika Rajput, Manjusha Pal, Manoj Pandey

**Affiliations:** Department of Surgical Oncology, Institute of Medical Sciences, Banaras Hindu University, Varanasi, 221005 India

## Abstract

A 60-year-old man presented with complaints of abdominal pain and melena. Patient had a history of colon cancer 16 years back and had undergone right hemi colectomy for microsatellite instability (MSI) negative, mismatch repair (MMR) stable, T2N0 disease with no mutations on next-generation sequencing (NGS). Investigations revealed a second primary in stomach (intestinal type of adenocarcinoma) with no recurrent lesions in colon or distant metastasis. He was started on CapOx with Bevacizumab and developed gastric outlet obstruction. Total gastrectomy with D2 lymphadenectomy and *Roux-en-Y* oesophageao-jejunal pouch anastomosis was done. The histopathology showed intestinal type of adenocarcinoma with pT3N2 disease. NGS showed 3 novel mutations in *KMT2A, LTK*, and *MST1R* gene. The pathway enrichment analysis and Gene Ontology were carried out, followed by the construction of protein–protein interaction network to discover associations among the genes. The results suggested that these mutations have not been reported in gastric cancer earlier and despite not having a direct pathway of carcinogenesis they probably act through modulation of host of miRNA’s. Further studies are needed to investigate the role of *KMT2A*, *LTK*, and *MST1R* gene in gastric carcinogenesis.

## Background

Gastric adenocarcinoma is the fifth most common cancer worldwide and the third deadliest cancer reported by GLOBOCAN [1]. Lauren classified gastric cancer based on the histomorphology as intestinal, diffuse, and intermediate types and these have different epidemiological and clinical features. Intestinal type of gastric cancer is a chronic multifactorial disease in which there is chronic inflammatory process pertaining to *H Pylori* infection persisting for years [2]. In 1992, Correa proposed the model of gastric carcinogenesis as chronic inflammation leading to metaplasia, dysplasia, and finally carcinogenic growth [3]. In the evolution various molecular changes occur during the process. Commonly involved pathways are the upregulation of group of molecular chaperones (upregulated HSP 70, HSP 90) which are commonly expressed in response to the stress, antiapoptotic pathway upregulation (BH3 interacting domain and downregulation of *TFF1, TFF2, REG 1A*, and *MAPK1* [4]. Diffuse type of gastric cancer is found in young patients which is generally associated with atrophic gastritis [2].

Other than histology, gastric cancers are classified according to molecular data, the cancer genome atlas (TCGA) consortium developed a molecular classification according to observed genetic alterations and grouped them into 4 subtypes, EBV positive subtype associated with Epstein Barr Virus infection, MSI type that show high frequency of microsatellite instability, genomically stable (GS) type due to *CDH1* gene leading to loss of proteins related to cell adhesion; and fourth with chromosomal instability (CIN) type associated with high number of somatic copy number alterations [5]. The frequently altered genetic mutations reported is *RTK/ MAPK* pathway alterations associated with *TP53* genetic mutations, which is most commonly associated with CIN subtype [6].

The molecular classification of gastric cancer based on genomics, transcriptomics, and proteomics has led to better understanding of the molecular pathogenesis of cancer and guides targeted therapies. Here we report a case where in three novel mutations were identified in a patient with history of colon cancer with second primary gastric cancer. Bioinformatic analysis at gene and protein level is presented to understand the pathways of carcinogenesis.

## Case presentation

A 60-year-old man presented to surgical oncology out patient with complaints of epigastric pain and melena for 1 month, patient had a history of colon cancer for which he had undergone extended right hemicolectomy 16 years back. The earlier colonic cancer was microsatellite (MSI) stable and mismatch repair (MMR) intact, with low tumour mutation burden (TMB) and no reported mutations on NGS. On clinical examination, ECOG performance status was 1, general examination was unremarkable, on abdominal examination, there was tenderness in the epigastrium however no mass/lump was palpable. There was no ascites, or organomegaly, rectal examination was unremarkable.

### Investigations

**Fig. 1:**
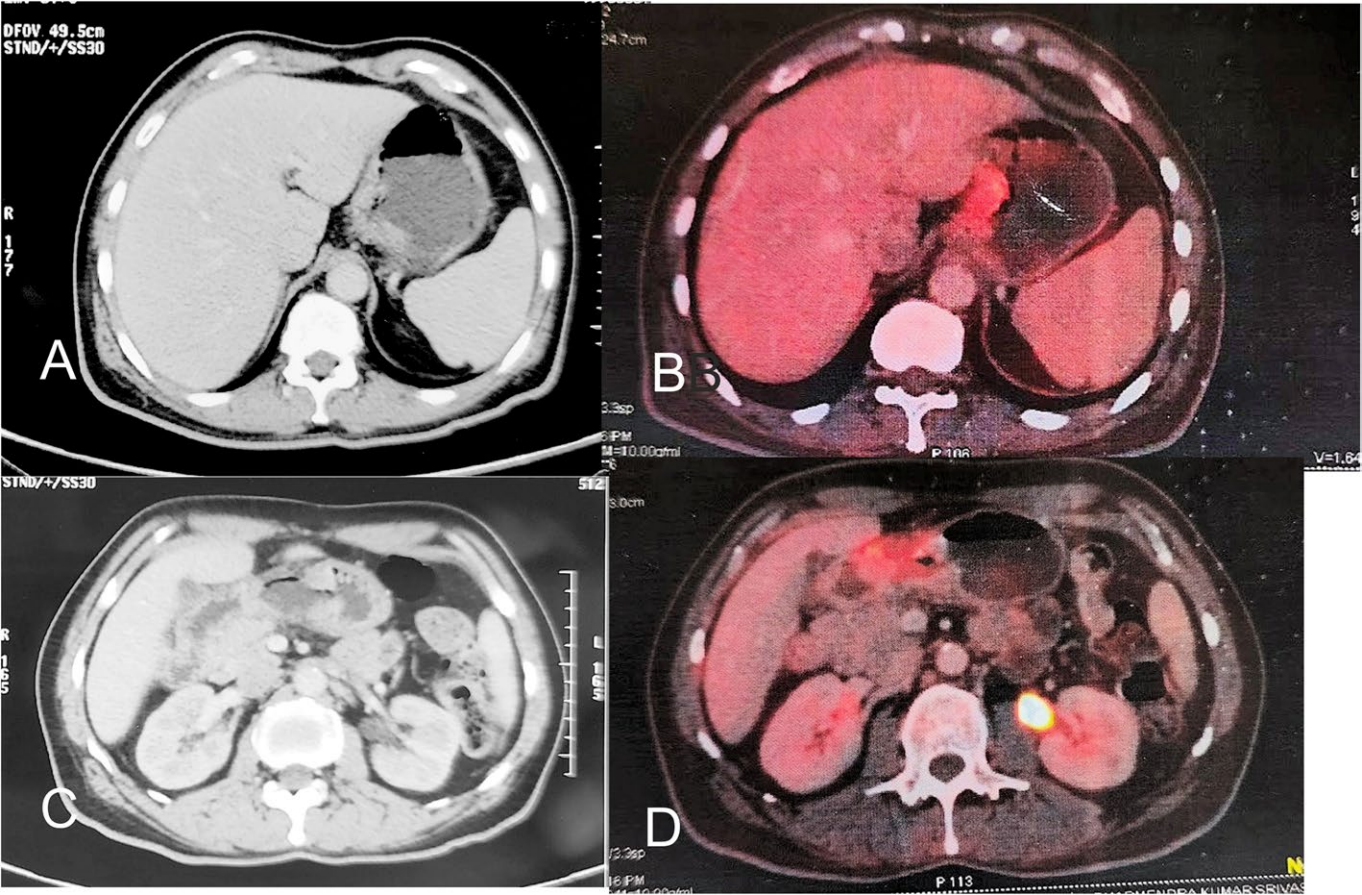
Contrast enhanced computerized tomography and PET scan showing lesions at cardia (**A**, **B**) and pylorus (**C**, **D**)

Routine haematology and biochemistry were within normal limits, contrast enhanced computed tomography (CECT) scan showed asymmetric eccentric gastric wall thickening, with few polypoidal lesions noted in gastroesophageal (GE) junction and antro-pyloric region with multiple sub centimetric lymph nodes along the lesser curvature and common hepatic artery, the plane with pancreas was obliterated (Fig. 1), the lesion was close to previous anastomotic site, however colon was free. An upper gastro intestinal endoscopy (UGI) showed a large ulceroproliferative growth (UPG) at gastro oesophageal junction extending to cardia of stomach, and another UPG at antro-pyloric area was seen with overlying unhealthy mucosa, a biopsy was taken. Keeping in mind his history of colonic cancer, a colonoscopy was performed that showed healthy anastomotic site with normal vascularity and mucosal pattern. Histopathology of the gastric biopsy suggested adenocarcinoma, immunohistochemistry (IHC) showed CDX2 and CD 20 positivity suggesting it to be intestinal type of adenocarcinoma. Positron emission tomography showed two FDG avid lesions in stomach one of which was close to previous anastomosis, there was no other lesion.

### Treatment

**Fig. 2:**
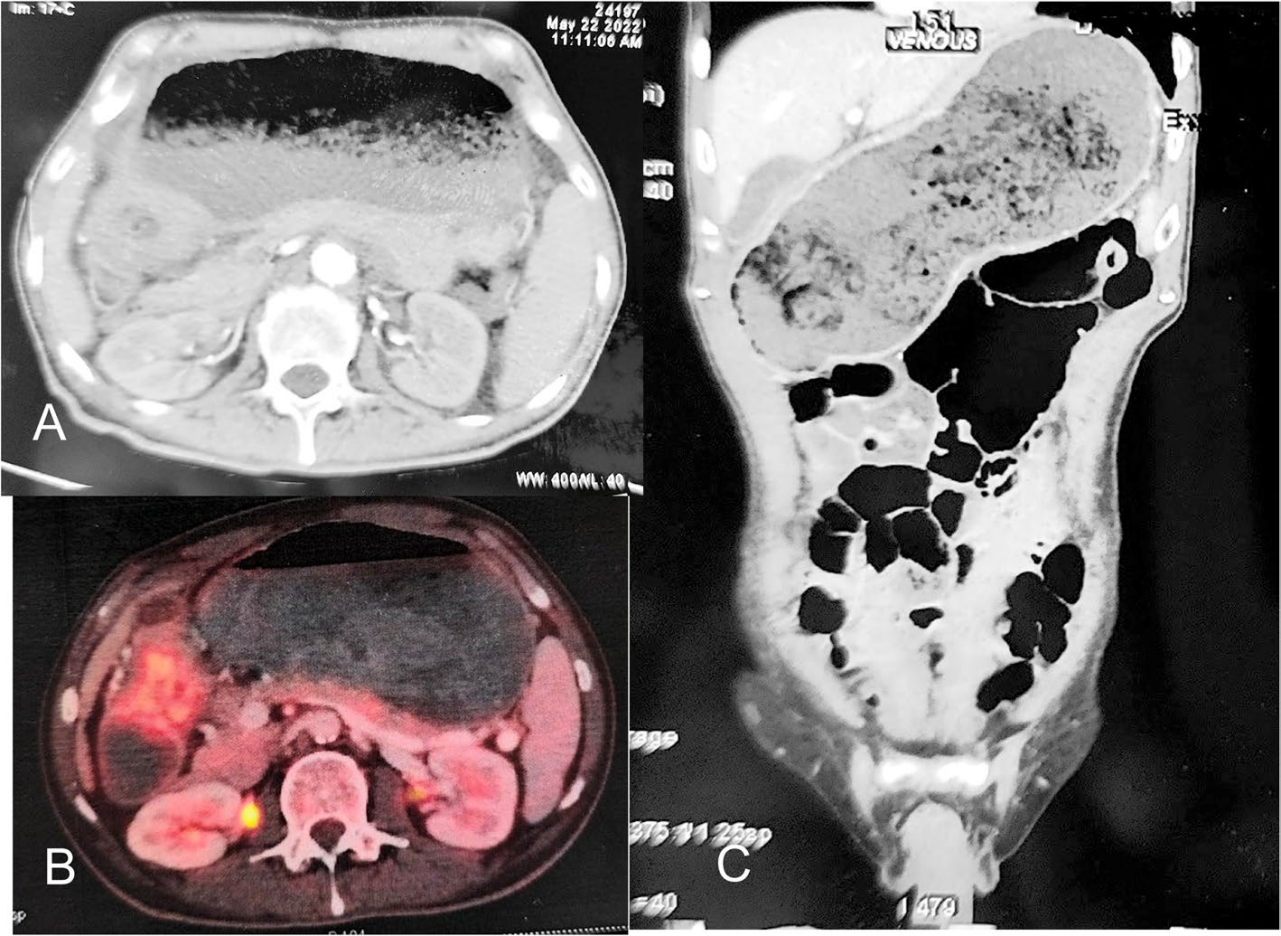
Contrast enhanced computerized tomography and PET scan showing pyloric obstruction (**A**, **B**), grossly distended stomach (**C**)

With an initial diagnosis of gastric metastasis from colon cancer, patient was started on oxaliplatin (100 mg/m^2^ 3 weekly), capecitabine (1000 mg/m^2^ twice a day, for 14 days with 1 week break), and bevacizumab (7.5 mg/m^2^ 3 weekly) as per institutional protocol. After 3 cycles of chemotherapy, repeat CECT showed a partial response and as patient developed features of peripheral neuritis oxaliplatin was stopped and he continued capecitabine and bevacizumab (CapBeva). After 7 cycles patient developed gastric outlet obstruction. Repeat imaging showed near complete obstruction of the pylorus with minimal residual disease, the plane with pancreas was still not clear (Fig. 2). Patient was taken up for surgery, intra operatively 3 × 3 cm lesion was seen with serosal involvement in gastroesophageal junction and cardia, another lesion was seen along the lesser curvature of stomach with serosal involvement, enlarged lymph nodes were seen in station 1 to 11. A total gastrectomy with D2 lymphadenectomy and Roux-en-Y oesophageao-jejunal pouch anastomosis was done. Postoperative period was uneventful, final histopathology showed moderately differentiated adenocarcinoma, intestinal type pT3N2. Patient was discharged on 10th postoperative day without any complications.

On IHC, HER2 was equivocal and amplification was negative on fluorescent in situ hybridization (FISH), PD-1 was immunoreactive with score 1 + in tumour infiltrating immune cells (IC), score 0 in tumour cells (TC), with ≥ 1% IC/TC-PDL1 (SP142 Ventana) expression. On NGS analysis, 3 novel mutations were identified. A missense mutation in exon 27 of *KMT2A* gene (p.Leu2824Pro) with mutant allelic burden 63%. A frame deletion in exon 7 of *LTK* gene (p.Ala321_Gly330del), with 29% mutant allelic burden, and another missense mutation in exon 17 of *MST1R* gene (p.His1206Arg) with mutant allelic burden of 40%. No targetable gene variants were detected, The TMB was low, and MMR, MI were stable.

After the surgery, the option of adjuvant chemotherapy was discussed with the patient however he refused further therapy due to weakness. He is alive and well 18 months after the surgery and has gained 7 kg weight.

### Bioinformatics analysis

To investigate the significance of mutations bioinformatic analysis was done using WEB-based Gene Set Analysis Toolkit (WebGestalt) (http://www.webgestalt.org/) to perform Gene Ontology (GO) and Functional pathway analysis. The *p* < 0.05 was chosen as the cut-off. NetworkAnalyst (http://www.networkanalyst.ca.) was used to construct a protein–protein interaction network and a signalling network among the stomach cancer-specific genes. GeneMania (https://genemania.org/) tool was utilized to construct a gene–gene interaction network.

**Fig. 3:**
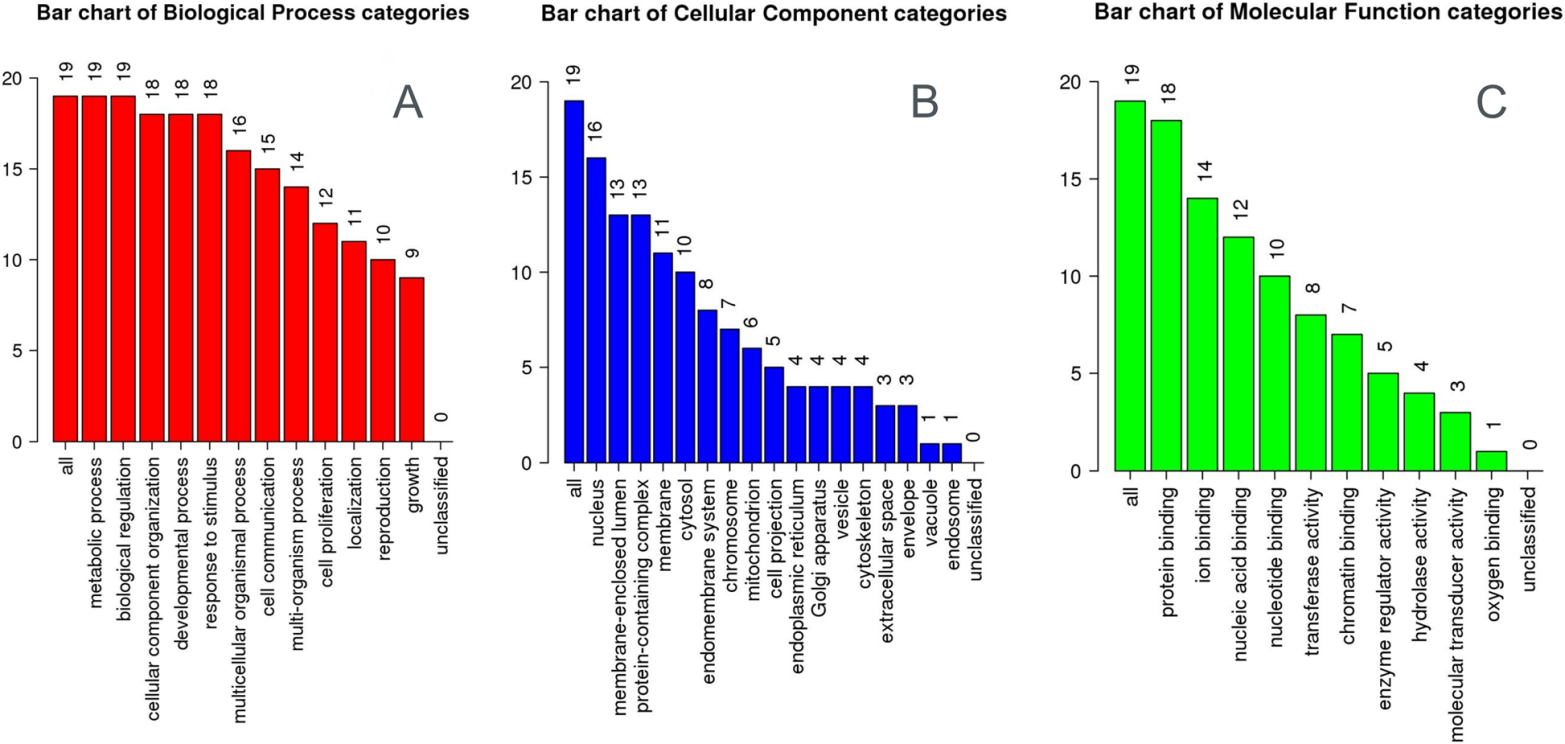
Gene Ontology through Webgestalt tool showing **A** biological process, b cellular components, and **C** molecular functions

The results of GO enrichment analysis were categorized into three functional categories, i.e., biological process (BP), molecular functions (MF), and cellular components (CC) (Fig. 3A–C). Three most common BP identified through these genes are metabolic process, development process, and response to stimulus; three most common CC are protein containing complexes and cellular membrane; and three most common MF are protein binding, transferase activity, and ion binding.

Reactome pathway enrichment analysis through webgestalt tool based on false discovery rate (FDR) showed eight positive and two negative categories (Table 1). The genes which are significantly enriched were *KMT2A* and *MST1R* gene.

**Table 1:** Reactome pathway enrichment analysis through Webgestalt Tool-

Gene set	Description	Size	Expect	Ratio	*P* value	FDR
R-HSA-8852405	Signaling by MST1	5	0.00094760	1055.3	0.00094742	1
R-HSA-3214841	PKMTs methylate histone lysines	71	0.013456	74.317	0.013411	1
R-HSA-8936459	RUNX1 regulates genes involved in megakaryocyte differentiation and platelet function	97	0.018383	54.397	0.018300	1
R-HSA-8939236	RUNX1 regulates transcription of genes involved in differentiation of HSCs	130	0.024638	40.588	0.024487	1
R-HSA-8878171	Transcriptional regulation by RUNX1	239	0.045295	22.077	0.044784	1
R-HSA-3247509	Chromatin modifying enzymes	275	0.052118	19.187	0.051441	1
R-HSA-4839726	Chromatin organization	275	0.052118	19.187	0.051441	1
R-HSA-9006934	Signaling by receptor tyrosine kinases	455	0.086231	11.597	0.084376	1
R-HSA-212436	Generic transcription pathway	1169	0.22155	4.5137	0.20929	1
R-HSA-73857	RNA polymerase II transcription	1292	0.24486	4.0840	0.22988	1

**Fig. 4:**
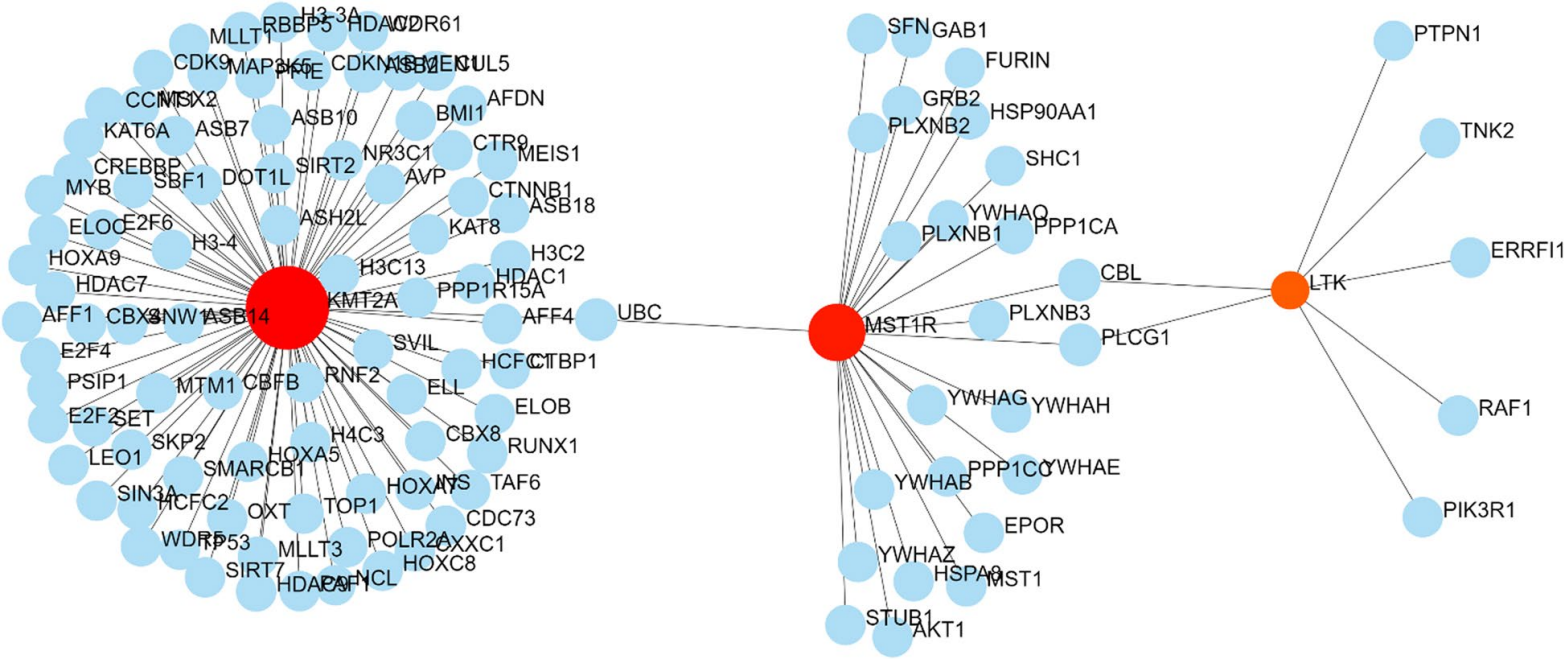
Protein–protein Interaction network through NetworkAnalyst using plugin IMEx Interactome showing network for KTM2A, LTK and MST1R genes

**Fig. 5:**
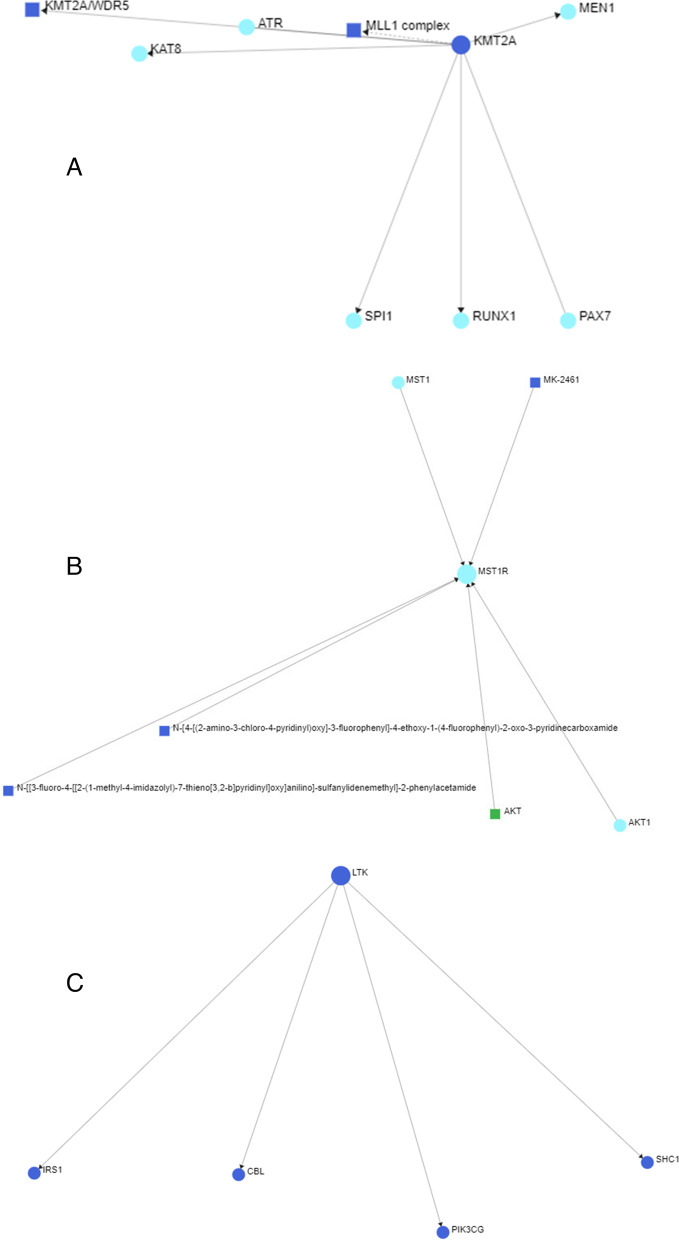
Signaling Interaction network through NetworkAnalyst showing network for KTM2A, LTK, and MST1R

**Fig. 6:**
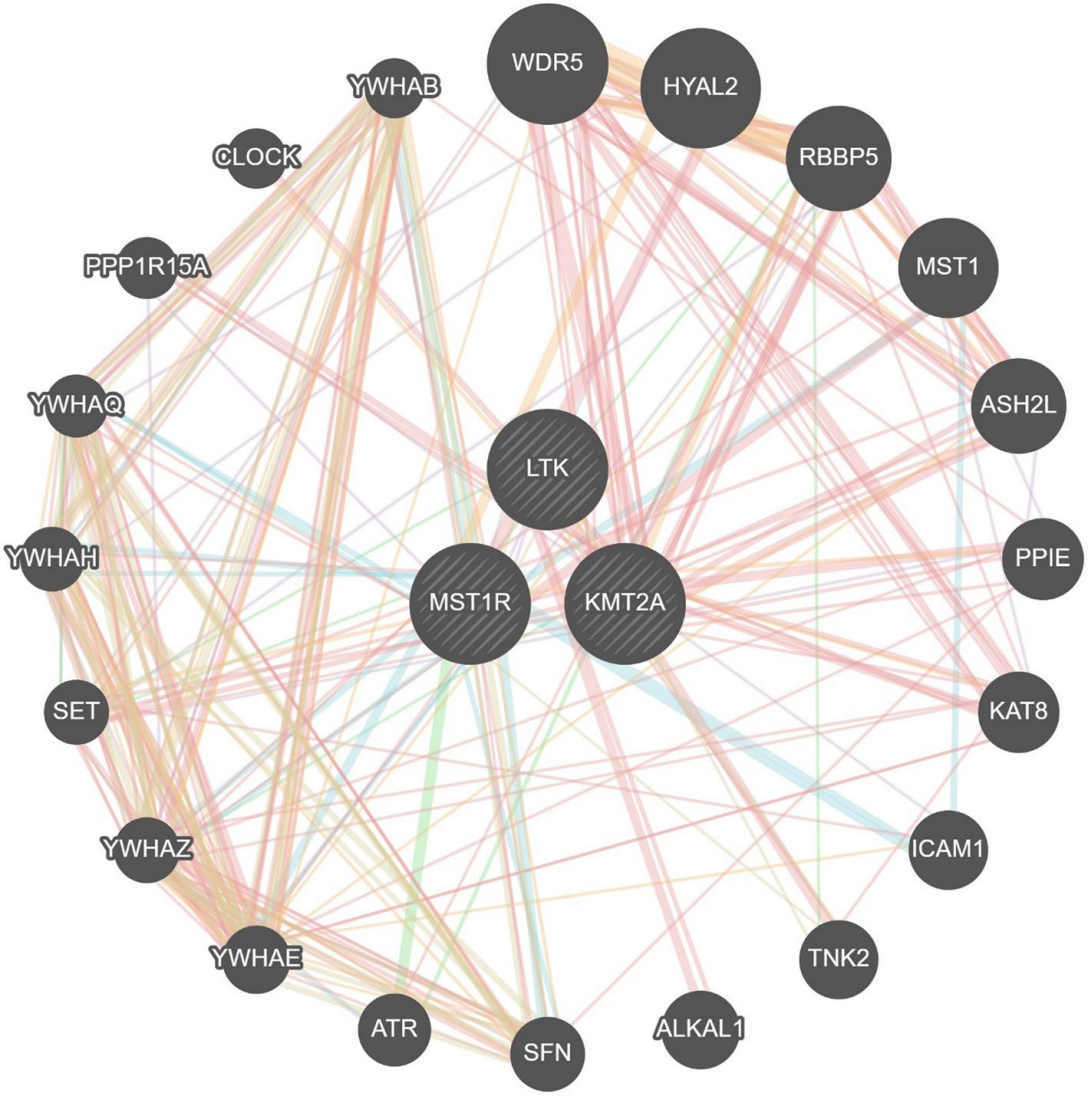
Gene–gene Interaction network through GeneMania

**Fig. 7:**
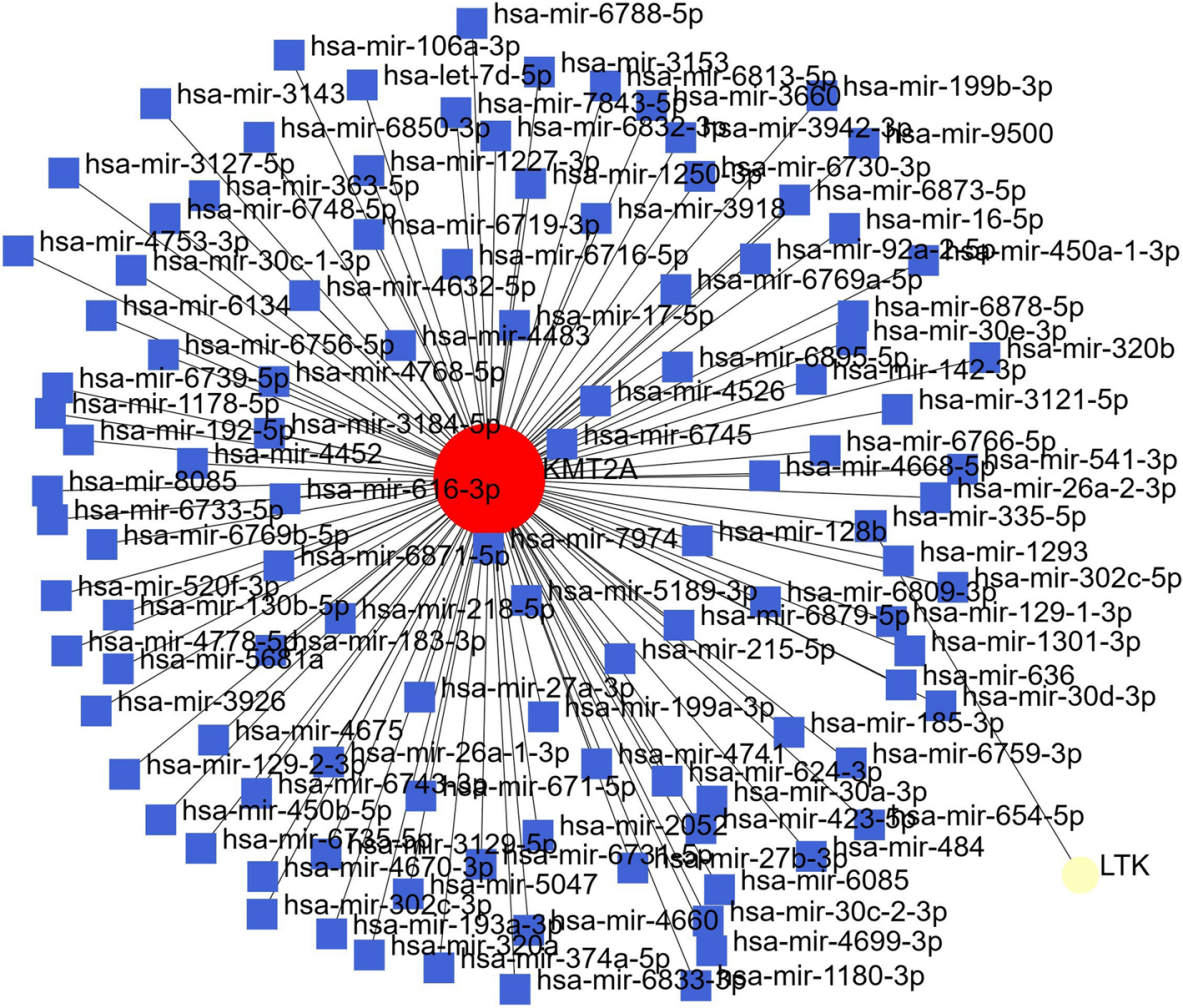
Gene-miRNA Interaction network through NetworkAnalyst for KTM2A and LTK gene showing interaction with number of miRNAs

Protein–protein Interaction network through NetworkAnalyst using plugin IMEx Interactome created, with 3 significant genes, had 3 nodes and 116 edges (Fig. 4), further signaling interaction network created through network analyst tool for all 3 genes separately through plugin Signor had 18 edges and 21 nodes (Fig. 5). Several hub genes exhibiting co-expression, predicted and physical and genetic interaction with other genes were identified, and a network was constructed through the gene mania tool (Fig. 6), Gene-miRNA Interaction network was prepared through NetworkAnalyst with miRTarBasev 8.0 with 2 genes, i.e., *KMT2A* and *LTK* with 113 nodes and 112 edges (Fig. 7).

## Discussion

Multi-omics characterisation of NGS data from cancer stomach identified 3 novel mutated genes and their functional and biological pathways in second primary gastric cancer in a patient with primary colon cancer with no mutations. To the best of our knowledge, *KTM2A, LTK*, and *MST1R* gene mutations are not reported in gastric cancer, thus the discovery of these 3 mutations is novel finding. Despite advances in diagnosis and treatment the 5-year survival of gastric cancer is only 20%, because the disease is detected late after the tumour has invaded muscularis propria, and in early stages only vague and non-specific symptoms are present [7], with the appropriate targeted therapy, prognosis of the patient can be improved.

*KTM2A* (lysine methyl transferase 2A) is a protein encoding gene on chromosome 11. It is a transcriptional coactivator that helps in haematopoiesis and neurodevelopment during embryogenesis. It has a ubiquitous expression in various tissues like lymph nodes, ovary, pancreas, and colon [8]. The germinal mutations of KTM 2A is associated with Wiedman-Stiener syndrome and of several chromatinopathies like Coffin–Siris syndromes, Kabuki syndrome, Cornelia De Lange syndrome, and Rubinstein–Taybi syndrome. Somatic mutations of this gene are responsible for acute lymphoid leukaemia and acute myeloid leukemias [9]. Pathogenic *KTM2A* mutation is not reported for colon cancer or gastric cancer.

*LTK* gene (leukocyte tyrosine kinase), is an endoplasmic reticulum resident receptor tyrosine kinase, which controls the secretion of endoplasmic reticulum (ER). Deletion of *LTK* is associated with reduction of ER sites and slow ER to Golgi body transport and thus regulate proteostasis [10], it is found to be overexpressed in human leukaemia. It shares around 80% of similarity in kinase domain with *ALK* (anaplastic lymphoma kinase) which is mutated in many human cancers like lymphomas and neuroblastomas [11]. *LTK* is expressed in hematopoietic cells of B cell lineages, placenta, duodenum, and stomach. *LTK* gene is not reported with any functional oncogenicity till now, but recently CLIP1-LTK fusion has been reported in non-small cell lung cancer [12].

*MST1R* (macrophage stimulating 1 receptor) gene is located on novel MECA 3 (major epithelial region affected region number 3) on chromosome 3p. It is a cell surface receptor for macrophage-stimulating protein (MSP) with tyrosine kinase activity. This region contains numerous unique gene that are responsible for vital cell functions and carcinogenesis. It is frequently affected in breast cancer, renal cell cancer, non-small cell lung cancer, and epithelial ovarian cancer [13].

Most common pathways seen in gastric carcinogenesis are the ErbB pathway with over expression or amplification of *ERRB1* (*EGFR*), *ERBB2* (*HER2*), *ERBB3* (*HER 3*) and *ERBB4* (*HER 4*) genes, among which only *HER2* overexpression is seen in 5–29.5% of the cases. Apart from that the *VEGF* over expression or amplification is seen in 54–82% of cases which is mostly in *VEGF-A*. *PI3K/AKT/mTOR* pathway shows overexpression of either PI3KCA, p-AKT, p-mTOR or p-P70S6K. Most common is p-AKT with expression in 40–80% of cases and p-mTOR most commonly overexpressed in 60% of intestinal type of cases. Next is *HGF/MET* pathway with over expression or amplification of *HGF* and *MET* is seen in 87% and 42% cases of gastric carcinogenesis respectively. Last pathway is Hedgehog pathway with overexpression of PTCH, SHH, SMO, IHH, and GLI1 seen in 16–69% of cases [14].

In our case, none of the above pathways were detected; however, three other novel pathogenic gene mutations were found. As these have not been reported in gastric cancer and their pathways are undiscovered we performed the bioinformatics to see the gene–gene and gene-protein interactions. KTM2A was found to have interactions with MEN1, MLL1 complex, ATR, KAT8, SP11, and PAX7 none of which have been implicated in gastric cancer before. Similarly, MST1R had interactions with AKT, AKT1, and MST1, while LTR interacted with IRS1, CBL, PIK3C and SHC1, none of which have been found to be associated with gastric cancer before. The KMT2A was found to interact with many miRNA, while LTK interacted with has-mir 335-5p, the findings suggest micro RNA regulation by these genes specifically by KMT2A and appears to be the primary mechanism through which KMT2A may induce carcinogenesis. Somatic mutation profiling in gastric cancer is necessary as it can shed light on newer pathways of carcinogenesis and can provide newer targets for treatment.

Here, we report unique novel mutations in three genes which were generally not associated with gastric cancer but may have carcinogenic potential, and hence are being reported. As no specific pathway is associated with the above-detected mutations; hence, no active targeted therapy can be recommended; however, they appear to regulate the miRNA. Further studies reporting on newer mechanisms and pathways in gastric cancer are necessary to personalize treatment for patients with gastric cancer.

## Data Availability

All data are provided in the manuscript.
